# Pulse vs. R-R Interval: Validity and Reliability of the Garmin Elevate v4 Sensor for Ultra-Short HR and HRV Monitoring in Healthy Adults

**DOI:** 10.3390/s26206549

**Published:** 2026-10-15

**Authors:** Antonio Casanova-Lizón, José Manuel Sarabia, Adolfo Aracil-Marco, Mario Martínez-García, Manuel Moya-Ramón, Agustín Manresa-Rocamora

**Affiliations:** 1 Sports Research Centre, Universidad Miguel Hernández, 03202 Elche, Spain; acasanova@umh.es (A.C.-L.); jsarabia@umh.es (J.M.S.); fito@umh.es (A.A.-M.); mario.martinez01@alu.umh.es (M.M.-G.); amanresa@umh.es (A.M.-R.); 2 Institute for Health and Biomedical Research of Alicante (ISABIAL), 03010 Alicante, Spain

**Keywords:** exercise prescription, heart rate variability, measurement agreement, photoplethysmography, wearable technology

## Abstract

**Highlights:**

**What are the main findings?**

- Wrist-based photoplethysmography (PPG) demonstrated near-perfect linear association, high concordance, and robust test–retest reliability for measuring resting heart rate across both supine and seated positions.
- While the smartwatch showed excellent intrasession test–retest reliability for PPG-derived PRV (rMSSD_PRV_), it exhibited a significant proportional bias and wide limits of agreement when compared with ECG-derived HRV.

**What are the implications of the main findings?**

- The study evaluated the Garmin Elevate v4 optical sensor against electrocardiography for resting heart rate and pulse rate variability (rMSSD_PRV_) against electrical heart rate variability (rMSSD_HRV_) measurements in healthy adults.
- Researchers and practitioners should not use optical PPG-derived PRV as a substitute for ECG-derived HRV for the assessment of resting autonomic function at the individual level.

**Abstract:**

Accurate resting heart rate (HR) and heart rate variability (HRV) monitoring is crucial for evaluating autonomic nervous system balance and guiding training. This study investigated the validity and reliability of the Garmin Forerunner 955 smartwatch (Elevate v4 sensor) against an electrocardiogram (ECG) reference for measuring resting HR and photoplethysmography (PPG)-derived pulse rate variability (PRV), evaluated through the root mean square of successive differences (rMSSD_PRV_), compared to reference ECG-derived HRV (rMSSD_HRV_). Twenty-three healthy adults underwent simultaneous assessments using the smartwatch and a Biopac ECG during short recordings in supine and seated positions under controlled breathing. For resting HR, the optical device demonstrated a near-perfect linear association (*r* ≥ 0.94) and substantial concordance with the ECG. While rMSSD_PRV_ demonstrated good-to-excellent intrasession test–retest reliability, its agreement with ECG-derived rMSSD_HRV_ was limited, showing significant proportional bias and wide limits of agreement. A significant proportional bias was detected, producing back-transformed limits of agreement that demonstrated relative measurement errors ranging from −43% to +52% in the supine position and from −41% to +118% in the seated position in isolated individual cases. Ultimately, the Garmin Elevate v4 is highly reliable for basic HR tracking and within-subject PRV trends, but it lacks the absolute accuracy required to replace reference ECG-derived HRV for individual resting autonomic evaluation.

## 1. Introduction

Heart rate variability (HRV), defined as the temporal fluctuation between consecutive heartbeats (R-R intervals), constitutes a widely established, non-invasive marker for assessing the balance of the autonomic nervous system (ANS) [1]. Alongside HRV, resting heart rate (HR) serves as a fundamental and complementary indicator of cardiovascular stress, overall training status, and physiological adaptation [2,3]. Among the various metrics available, the root mean square of successive differences (rMSSD) emerges as the most reliable and practical index for quantifying resting parasympathetic modulation. This preference is supported by evidence indicating that rMSSD is more statistically robust and less influenced by respiratory rate and other methodological factors compared to frequency-domain indices [4,5]. Although 5-min recordings have traditionally been used to measure HRV, recent evidence suggests that ultra-short HRV recordings can provide acceptable estimates of RMSSD under controlled resting conditions, although their validity and reliability may depend on recording duration, measurement conditions, and the specific HRV metric assessed [6,7,8]. For instance Esco et al. [7] and Moya-Ramon et al. [8] reported near-perfect correlations and minimal bias between 1-min recordings and traditional timeframes, thereby greatly facilitating athletes’ adherence to daily monitoring under stable conditions.

For the acquisition of these data, the electrocardiogram (ECG) is universally considered the gold standard [9]. Nevertheless, in the sporting context, chest straps have proven to be a highly rigorous alternative. Specifically, devices such as the Polar H10 detect the heart’s electrical signal with an accuracy that reproduces ECG-derived HRV with near-perfect correlation (*r* > 0.99) and negligible bias under resting conditions [10]. Given this overwhelming evidence, the chest strap is accepted in contemporary scientific practice as a valid reference device for stationary HRV measurement.

Concurrently, technological accessibility has driven a paradigm shift towards the use of photoplethysmography (PPG) via smartwatches and mobile devices. It is crucial to distinguish that whilst the ECG and chest straps measure electrical events (R-R intervals), PPG detects mechanical changes in peripheral blood volume. The signal derived from this method is termed pulse rate variability (PRV) [11]. Although pulse transit time can introduce technical discrepancies between PRV and HRV, particularly for time-domain metrics such as rMSSD, recent evidence indicates that the agreement between PPG-derived PRV and ECG-derived HRV may depend on factors such as body position, recording duration, and sensor characteristics [12,13]. Nevertheless, PPG-derived PRV can provide a useful surrogate estimate of HRV under controlled resting conditions. However, the validity of measurement of PPG is strongly influenced by sensor placement, postural changes, and device technology [14,15,16]. In this regard, a recent validation study indicates that the accuracy of consumer wearables varies substantially according to device and measurement context, with some devices showing stronger agreement with ECG during nocturnal monitoring than others [17].

Within the specific field of sports performance, Garmin has introduced features such as the Health Snapshot and the continuous measurement of nocturnal HRV status. Authors such as Williams et al. [18] have reported strong agreement with ECG when these features were evaluated under controlled (“laboratory-based”) conditions. Nevertheless, a notable gap remains in the literature regarding the independent validation of latest-generation optical sensors, such as the Elevate v4, particularly under conditions and using commercially available ultra-short measurement protocols, such as the 2-min Health Snapshot, against an ECG gold-standard. Before establishing its utility in dynamic day-to-day use scenarios, it is mandatory to determine its baseline accuracy under highly controlled, standardized resting conditions.

To effectively guide training periodisation, the precise monitoring of HRV must be conducted strictly under resting conditions, typically in the morning immediately upon waking [5]. Although evidence regarding performance outcomes remains mixed, recent interventions suggest that HRV-guided training may improve selected physiological outcomes while reducing high-intensity training volume in specific populations and training contexts [19,20,21]. For this methodology to be practically applicable, it is imperative to utilise highly accurate and validated measurement tools. Therefore, the primary objective of this study was to evaluate the concurrent validity and reliability of the Garmin Forerunner 955 smartwatch, equipped with the Elevate v4 optical sensor, for measuring resting HR and PPG-derived PRV (i.e., rMSSD_PRV_) against the ECG for electrical HRV (i.e., rMSSD_HRV_) in healthy adults. Specifically, evaluations were conducted in both supine and seated positions to ascertain whether orthostatic shifts and subsequent changes in peripheral blood volume influence the accuracy of the optical sensor.

## 2. Materials and Methods

### 2.1. Study Design

An observational, cross-sectional validation and methodological agreement study was conducted. The design and reporting of this study followed the GRRAS (Guidelines for Reporting Reliability and Agreement Studies). Two acquisition methods were evaluated simultaneously: (1) a Biopac MP35 electrocardiograph (Biopac Systems, Goleta, CA, USA); and (2) a Garmin Forerunner 955 (Garmin Ltd., Olathe, KS, USA) recording via PPG through its Elevate v4 wrist-based optical sensor.

### 2.2. Participants

A cohort of healthy adult volunteers, aged between 20 and 45 years, was prospectively recruited to participate in this investigation. Regarding eligibility, inclusion required participants to be entirely free from known cardiovascular pathologies or ANS disorders. Conversely, exclusion criteria comprised individuals undergoing any pharmacological treatment capable of altering cardiac autonomic responses. The sample size was statistically justified based on the minimum requirements for reliability studies. According to Walter et al. [22] and Bujang and Baharum [23], to detect a minimum acceptable intraclass correlation coefficient (ICC) of 0.80 against a null hypothesis of 0.50 with an alpha of 0.05 and 80% power across two repeated measurements, a minimum of 22 participants is required. Therefore, our cohort of 23 participants provided adequate statistical power for the primary reliability endpoints. This sample size also aligns with robust precedents established in previous wearable validation research [8,24].

Prior to enrolment, all participants provided written informed consent. Furthermore, participants were instructed to abstain from consuming caffeine or alcohol, and from engaging in strenuous physical exercise during the 24 h prior to laboratory testing, strictly adhering to standardised guidelines for short-term HRV measurement [25]. The experimental protocol was formally approved by the Institutional Ethics Committee of the Miguel Hernández University (Code: DCD.DPC.02.23-01.19). The study was conducted in strict accordance with the ethical principles mandated by the Declaration of Helsinki [26].

### 2.3. Experimental Setting and Data Acquisition

Assessments were conducted in a laboratory under strictly controlled environmental conditions (temperature of 21–22 °C, constant relative humidity, and acoustic isolation). To minimise the influence of respiratory sinus arrhythmia (RSA) on vagal modulation, participants were instructed to maintain a paced breathing rate of 12–15 breaths per minute (0.20–0.25 Hz) guided by an auditory metronome, a widely recommended procedure to standardise short-term HRV recordings [15,27].

The Biopac MP35 system (considered the reference standard) captured the ECG signal at a sampling frequency of 1000 Hz using the Biopac Student Lab software (v. 3.7.1) [28,29]. Simultaneously, the Garmin (firmware version 27.09) device was utilised for wrist-based PPG and operated via the Health Snapshot function using its default settings. Given that its native algorithm does not permit the temporal export of pulse-to-pulse intervals, the raw HR and PPG-derived PRV (rMSSD_PRV_ in ms) values displayed on the screen were manually recorded immediately following each 2-min assessment. The numerical resolution of the displayed metrics was 1 beat per minute (bpm) for HR and 1 ms for rMSSD_PRV_. To strictly prevent transcription errors, all recorded data were immediately entered into a digital database and independently double-checked by a second researcher. Furthermore, the investigator responsible for reading and recording the Garmin outputs was inherently blinded to the reference ECG results, as the raw ECG signals required subsequent offline processing and filtering in Kubios HRV software to compute time-domain indices. Finally, all statistical analyses were conducted by an independent researcher who was fully blinded to participant identities and data collection conditions, working exclusively with anonymised dataset codes.

### 2.4. Procedures

Following skin preparation (cleansing with alcohol and mild dermal abrasion to reduce impedance), the surface electrodes (Ag/AgCl) for the Biopac system were placed according to the modified Central Sub-clavicular 5 (CS5) three-lead configuration. This arrangement was selected because its measurement axis traverses the greatest muscle mass of the left ventricle, which significantly maximises the amplitude of the R wave and minimises baseline artefacts, thereby optimising the automatic detection of R-R intervals by the software [30,31]. Anatomically, the negative electrode was positioned in the right sub-clavicular region, the positive electrode was placed in the equivalent V5 position (fifth left intercostal space, on the anterior axillary line), and the reference electrode was located in the right iliac fossa (Figure 1).

Subsequently, the watch for PPG assessment was fitted to the left wrist, ensuring a firm adjustment immediately proximal to the ulnar styloid process. This guaranteed stable contact between the optical sensor and the skin without generating excessive compression that could alter local perfusion [32,33].

The protocol consisted of four consecutive 3-min and 30-s recordings, alternating between supine and seated positions, as postural changes significantly alter venous return and sympathovagal balance [34]. Each block included 1 min of stabilisation followed by 2 min and 30 s of recording, adhering to the Task Force standards [35]. Due to the operational requirements of the Health Snapshot function, the initiation of synchronisation between devices was performed manually by the researcher. The PPG sensor recording was manually initiated with an exact 30-s delay relative to the start of the ECG recording (at t = 1:30), capturing an ultra-short 2-min (120-s) window (Figure 2) [6,7]. Both devices recorded simultaneously until t = 3:30, ensuring identical start and end times were strictly maintained for each recording.

All measurements were conducted by the same investigator, and the recordings were supervised simultaneously across both devices. In cases where synchronisation errors (e.g., during the manual initiation) or artefacts were detected during recording, the protocol was repeated [36]. Measurements were carried out individually for each participant.

Note on graphical representations: The conceptual diagrams illustrating the anatomical placement of the electrodes (Figure 1) and the experimental protocol timeline (Figure 2) were initially drafted using ChatGPT (version GPT-5.6, OpenAI) based on explicit text prompts provided by the authors. The AI-generated baseline images were strictly used for foundational layout and were subsequently reviewed, corrected, and manually annotated by the research team using standard image editing software to ensure precise anatomical and methodological accuracy.

### 2.5. Data Processing

The ECG R-R interval files (.acq) were then processed using the specialised Kubios HRV Premium software (version 4.3.0, Kubios Oy, Kuopio, Finland), which is considered the current gold standard for HRV analysis [37]. An automatic medium-level artefact correction filter (0.25 s) based on cubic spline interpolation was applied [38], which is particularly recommended for the treatment of occasional artefacts and ectopic beats in R-R interval analysis [39]. Only recordings with fewer than 20% corrected beats were included, adhering to the criterion of preserving at least 80% of consecutive normal beats to ensure reliable estimates of HRV indices [40,41]. The rMSSD metric was extracted for both devices to yield ECG-derived rMSSD_HRV_ and PPG-derived rMSSD_PRV_. Due to the asymmetric distribution of time-domain indices, absolute values were logarithmically transformed (LnrMSSD_HRV_ and LnrMSSD_PRV_) [42].

### 2.6. Statistical Analysis

Statistical analysis was performed using STATA software (version 16.0; StataCorp LLC, College Station, TX, USA). Descriptive data are presented as mean ± standard deviation (SD). Sample distribution normality for HR, ECG-derived LnrMSSD_HRV_, and PPG-derived LnrMSSD_PRV_ was confirmed via the Shapiro–Wilk test [43].

To satisfy the assumption of data independence, only the first trial of each posture (supine and seated) was entered into the concurrent validity and absolute agreement analyses (Pearson correlation, Lin’s concordance correlation coefficient, linear regression, and Bland–Altman plots). Both repeated measurements (Trial 1 and Trial 2) were utilised exclusively for assessing intrasession test–retest reliability.

The degree of linear association between the devices was determined using the Pearson correlation coefficient (*r*). The magnitude of these correlation coefficients was interpreted according to the guidelines proposed by Hopkins et al. [44]: <0.1 (trivial), 0.1–0.29 (small), 0.3–0.49 (moderate), 0.5–0.69 (high), 0.7–0.89 (very high), and ≥0.9 (near perfect).

To assess concordance between the Garmin PPG and ECG measurements, Lin’s concordance correlation coefficient (CCC, *p_c_*) was calculated. The CCC incorporates both a measure of precision and a bias-correction factor (*C_b_*), thereby quantifying departure from perfect concordance. The magnitude of the CCC was interpreted based on Akoglu [45] criteria: <0.90 (poor), 0.90–0.94 (moderate), 0.95–0.99 (substantial), and >0.99 (near perfect). Furthermore, the Bland–Altman graphical method was applied to determine systematic bias (mean difference) and 95% limits of agreement (LoA, ±1.96 × SD) [46], allowing for a visual examination of the error distribution. Linear regression analysis was applied to the Bland–Altman data to check for the presence of proportional bias.

Relative reliability was evaluated using the ICC. A two-way mixed-effects model, single measurement, with absolute agreement was utilised. ICC magnitudes were interpreted in accordance with Portney and Watkins [47] as poor to moderate (<0.75), good (0.75–0.90), and excellent (>0.90). Absolute reliability and random measurement error were quantified via the standard error of measurement (SEM) and the coefficient of variation of standard error of measurement (CV_SEM_) [48]. Subsequently, the minimal detectable change (MDC) was calculated using the formula MDC = 1.96 × SEM × √2, establishing the statistical threshold required to identify true physiological changes beyond the device’s inherent measurement error.

Finally, mean absolute error (MAE) and mean absolute percentage error (MAPE) were calculated to characterise the magnitude of measurement error across repeated trials and to complement the reliability analysis [49].

To evaluate clinical and practical acceptability a priori, predefined error thresholds were established based on sports medicine guidelines. For resting HR, a MAPE < 5% and Bland–Altman limits of agreement within ±5 bpm was defined as clinically acceptable [50,51]. For PPG-derived PRV (LnrMSSD_PRV_), acceptable concurrent validity against the reference ECG-derived HRV (LnrMSSD_HRV_) was defined as the device error remaining within the typical daily biological variation threshold (≈10–12%), while intrasession reliability was considered acceptable if the MDC remained below this biological noise [3,52].

The level of statistical significance for all tests was set a priori at *p* < 0.05. Furthermore, 95% confidence intervals (CI) were also reported.

## 3. Results

### 3.1. Baseline Cohort Characteristics

A total of 23 healthy adult participants (12 men and 11 women) successfully completed the entire experimental protocol without any dropouts or technical data losses. The detailed anthropometric, demographic, and baseline physiological characteristics are summarised in Table 1.

### 3.2. Electrocardiogram Signal Processing and Artefact Correction

Across all 92 completed ECG recordings (23 participants across 4 conditions), the mean proportion of corrected beats was 0.11% (range: 0.00–4.26%), with 94.6% of all recordings (87 out of 92) requiring no beat correction. Detailed beat counts and correction metrics for each participant and condition are provided in Tables S1–S4 (Supplementary Material). Because the maximum correction rate in any single recording (4.26%) was well below the predefined 20% threshold, no recordings were excluded, and sensitivity analyses regarding artifact correction were not required.

### 3.3. Validity and Reliability of PPG-Derived PRV (LnrMSSD_PRV_)

The validity results comparing the Garmin PPG-derived (LnrMSSD_PRV_) against reference ECG-derived HRV (LnrMSSD_HRV_) are detailed in Table 2. In the supine position, PPG-derived LnrMSSD_PRV_ and reference ECG-derived LnrMSSD_HRV_ demonstrated a near-perfect linear association (*r* = 0.92 [95% CI = 0.81, 0.96]; Figure 3A). Furthermore, overall concordance was classified as poor (*p_c_* = 0.89 [95% CI = 0.80, 0.97]), despite a high bias correction factor (*C_b_* = 0.97). The Bland–Altman graphical analysis confirmed a minimal systematic bias (–0.07 [95% CI = −0.18, 0.04]), demonstrating a negligible tendency for the optical device to underestimate the metric compared to the reference standard (Figure 4A). Regarding random error dispersion, the 95% LoA ranged from −0.56 [95% CI = −0.75, −0.37] to 0.42 [95% CI = 0.23, 0.60]. However, translating these limits into relative percentage errors (from −43% to +52%) highlights that extreme discrepancies between PRV and HRV can occur in isolated individual cases. A linear regression analysis of the difference against the mean revealed a statistically significant negative proportional bias (*β* = −1.36 [95% CI = −2.11, −0.61]; *p* < 0.01; Figure 4A).

During the seated evaluation, the linear association between PPG-derived LnrMSSD_PRV_ and reference ECG-derived LnrMSSD_HRV_ remained very high (*r* = 0.83 [95% CI = 0.63, 0.93]; Figure 3B), albeit with similarly poor concordance (*p_c_* = 0.78 [95% CI = 0.64, 0.93]; *C_b_* = 0.95). The Bland–Altman method yielded a bias of 0.13 [95% CI = −0.02, 0.27], reflecting a slight overestimation by the Garmin PPG (Figure 4B). In terms of random error, the 95% LoA expanded from −0.52 [95% CI = −0.77, −0.27] to 0.78 [95% CI = 0.53, 1.02]. As with the supine data, these substantial relative errors (ranging from −41% to +118%) indicate the potential for considerable discrepancies between PRV and HRV when used for individual assessments. A statistically significant negative proportional bias was also found (*β* = −0.95 [95% CI = −1.46, −0.44]; *p* < 0.01; Figure 4B).

The test–retest reliability results across repeated trials (Trial 1 vs. Trial 2) for both PPG-derived PRV (LnrMSSD_PRV_) and ECG-derived HRV (LnrMSSD_HRV_) are presented in Table 3. In the supine position, the Garmin PPG device demonstrated intra-subject consistency identical to that of the gold standard, with both achieving an ICC of 0.84. The optical sensor exhibited notable temporal stability, a MAE of 0.19, a MAPE of 4.72%, and a CV_SEM_ of 3.07%. Meanwhile, the ECG measurements for HRV showed a MAE of 0.23, a MAPE of 5.55%, and a CV_SEM_ of 4.28%. Furthermore, the MDC was established at 0.36 for the optical device and 0.50 for the ECG.

During the seated evaluation, the ECG-derived HRV showed superior reliability (ICC = 0.96) compared to the consistency observed for PPG-derived PRV with the Garmin sensor (ICC = 0.79). Nevertheless, the wearable sensor maintained a highly constrained level of variability for PRV (LnrMSSD_PRV_), reporting a MAE of 0.21, a MAPE of 4.73%, and a CV_SEM_ of 3.43%, while reference ECG-derived HRV (LnrMSSD_HRV_) recorded a MAE of 0.14, a MAPE of 3.51%, and a CV_SEM_ of 2.09%. Furthermore, the MDC was established at 0.42 for the optical device and 0.25 for the ECG. This ensures a sufficiently narrow margin of error to guarantee adequate reproducibility of successive PRV measurements.

### 3.4. Validity and Reliability of PPG-Derived Heart Rate

The validity results for HR comparing the Garmin PPG sensor and the ECG are detailed in Table 4. In the supine position, both systems exhibited a near-perfect linear association (*r* = 0.99 [95% CI = 0.96, 0.99]; Figure 5A). The regression slope was optimal (1.00; Figure 5), indicating no evidence of proportional bias across the observed range of HR. Accordingly, overall concordance was substantial (*p_c_* = 0.98 [95% CI = 0.97, 0.99]), supported by a high bias-correction factor (*C_b_* = 0.99), indicating little departure from perfect concordance. The Bland–Altman graphical analysis yielded a systematic bias of 0.52 [95% CI = −0.12, 1.16] bpm, confirming a marginal and clinically irrelevant overestimation by the optical sensor compared to the standard (Figure 6A). Regarding random error dispersion, the 95% LoA were narrow and clinically acceptable, ranging from −2.38 [95% CI = −3.48, −1.27] to 3.41 [95% CI = 2.31, 4.52] bpm. Additionally, no statistically significant proportional bias was found (*β* = 0.86 [95% CI = −1.21, 2.93]; *p* = 0.40; Figure 6A).

During the seated evaluation, the strength of the linear association remained near-perfect (*r* = 0.94 [95% CI = 0.85, 0.97]; Figure 5B), alongside with moderate concordance (*p_c_* = 0.93 [95% CI = 0.88, 0.99]; *C_b_* = 0.99). The Bland–Altman method reported a bias of −0.55 [95% CI = −1.84, 0.73] bpm, reflecting a slight underestimation of HR by the Garmin PPG (Figure 6B). In terms of random error, the 95% LoA expanded from −6.38 [95% CI = −8.61, −4.16] to 5.28 [95% CI = 3.05, 7.51] bpm. The magnitude of the error and the inherent discrepancy of the device proved to be negligible compared to the strict accuracy demands required in exercise physiology, thereby consolidating the instrument’s validity. Moreover, no statistically significant proportional bias was found (*β* = 0.78 [95% CI = −0.51, 2.08]; *p* = 0.22; Figure 6B).

Regarding HR, the stability of repeated measures proved excellent for both instruments across all postures (Table 5). In the supine position, the Garmin PPG and the ECG presented virtually identical consistency levels (ICC of 0.92 and 0.93, respectively). The error analysis for the optical sensor reflected a MAE of 3.78 bpm, a MAPE of 6.33%, and a CV_SEM_ of 4.19%, values closely aligned with those of the reference standard, which reported a MAE of 4.31 bpm, a MAPE of 7.37%, and a CV_SEM_ of 4.12%. Finally, the MDC was set at 6.68 bpm for the Garmin and 6.57 bpm for the ECG.

The seated position showed similarly high reproducibility, with the Garmin device maintaining an ICC of 0.92. Furthermore, the optical sensor exhibited a MAE of 3.35 bpm, a MAPE of 5.23%, and a CV_SEM_ of 3.43%. These metrics were highly comparable to those demonstrated by the ECG (MAE = 3.18 bpm; MAPE = 5.10%; CV_SEM_ = 3.18%). Finally, the MDC was set at 5.82 bpm for the Garmin and 5.43 bpm for the ECG.

## 4. Discussion

The objective of this study was to determine the validity and reliability of the Garmin Forerunner 955 (Elevate v4) smartwatch for assessing HR and its variability (rMSSD) in the supine and seated positions in healthy adults. Although the device demonstrates a near-perfect linear association for resting HR, its capacity to estimate optical PRV (rMSSD_PRV_) reveals a significant proportional bias and critically wide LoA when compared to ECG-derived (rMSSD_HRV_). Paradoxically, it exhibits outstanding temporal (test–retest) reliability, highlighting a marked biostatistical dissociation between its internal consistency and clinical validity.

Since the seminal work of Bland and Altman [46], biostatistics has strongly cautioned against validating clinical instruments based solely on mean comparisons (ANOVA) or Pearson correlations. Mathematically, if a sensor overestimates HRV in certain participants whilst underestimating it in others, these opposing errors cancel each other out in the aggregate mean. This generates a ‘false positive’ of validity that masks severe individual instability. Physiologically, this bias stems from the inherent nature of the signal: ECG detects ventricular depolarisation, whereas PPG assesses PRV by measuring peripheral volumetric changes. In response to heightened parasympathetic tone, the pulse transit time becomes unstable due to fluctuations in arterial compliance [53]. This micromechanical lag remains uncorrected by the wrist-based algorithm, thereby amplifying the error in participants with high vagal tone.

In alignment with our predefined acceptability criteria, the optical device met the predefined acceptability criteria for resting HR and demonstrated acceptable intrasession reliability for PPG-derived PRV (LnrMSSD_PRV_). However, for LnrMSSD concurrent validity against ECG-derived HRV (LnrMSSD_HRV_), individual agreement fell well outside acceptable boundaries. To fully appreciate the practical implications of these findings, the agreement analysis should be interpreted on a relative percentage scale by back transforming the logarithmic LoA. In the supine position, the Bland–Altman limits ranged from −0.56 to 0.42. Back transforming these boundaries yields multiplicative ratios of 0.57 and 1.52. Applying the conversion formula (100 × (e^x^ − 1)) [46,54], this translates to relative percentage errors ranging from −43% to +52%. In the seated position, the limits ranged from −0.52 to 0.78, yielding multiplicative ratios of 0.59 and 2.18. Using the same mathematical conversion for the upper limit, for instance (100 × (e^0.78^ − 1) ≈ 118%), this represents relative errors from −41% to +118%. Accordingly, a single optical measurement may differ substantially from the ECG-derived HRV value for a given individual across both postures. According to Hopkins [54], measurement error of this magnitude exceeds the normal day-to-day biological variation in resting vagally mediated HRV (~10–12%), thereby reducing the ability to distinguish genuine physiological adaptations from analytical noise [3,5,55]. Likewise, Atkinson and Nevill [56] emphasised that the practical interchangeability of two measurement methods should be judged primarily according to the magnitude of their LoA to the intended physiological application.

Beyond concurrent validity, the short-term utility of a wearable device relies upon its intraday reliability. In this regard, the Garmin Elevate v4 exhibits outstanding absolute reliability. The MAPE remained below the 5% excellence threshold, and the CV_SEM_ was approximately 3.5%. Furthermore, in the supine position, the MDC of the optical sensor (0.36) surpassed even the ECG gold standard (0.50) in temporal consistency.

This illustrates a classic paradigm in sports data acquisition systems: the device demonstrated high within-device reliability, whereas its proportional bias and wide limits of agreement indicated limited agreement with ECG-derived HRV. Ultimately, whilst the watch is consistent across repeated measurements (thus successfully capturing trends), its PRV measurements showed limited agreement with ECG-derived HRV for estimating the absolute magnitude of rMSSD under the conditions tested.

Our findings align with prior Garmin validation studies. For example, Theurl et al. [57] found high HR agreement between the Garmin vívoactive 4 and ECG, yet poorer accuracy for HRV metrics like rMSSD. This mirrors our results showing excellent HR agreement but wider limits of agreement for LnrMSSD_PRV_, particularly during seated rest. Broader literature confirms that wearable-derived HRV accuracy fluctuates significantly across platforms. Specifically, HRV agreement varies widely among devices including the Garmin Fenix 6 [17], Apple Watch, and Oura Ring under differing measurement [58,59,60]. Recent wrist-worn PPG validations similarly demonstrate that ECG agreement is metric-dependent [61]. Consequently, the validity of wearable-derived HRV must be assessed individually for each specific index rather than broadly extrapolated. Furthermore, sensor anatomical placement critically influences ECG agreement. Finger-based capillary PPG sensors typically yield narrower HRV limits of agreement than wrist-worn alternatives during rest [36]. This likely reflects local differences in pulse-wave transmission and signal quality, although the precise contribution of tissue characteristics cannot be deduced from the present study. Collectively, good validity HR does not necessarily imply comparable validity for HRV when both are derived from the same PPG device. Rigorous model and measurement-site-specific validation remains essential before substituting wearable-derived HRV for gold-standard ECG measurements.

Compared with fully optical systems, hybrid approaches offer greater analytical accuracy. Sports prescription platforms such as Selftraining UMH [24] address the inherent limitations of peripheral PPG by electrically extracting R-R intervals through Bluetooth connectivity with validated chest straps such as the Polar H10. Consequently, the cardiac electrical signal is captured directly, avoiding inaccuracies related to pulse transit time. Alternatively, smartphone camera-based applications (e.g., HRV4Training [62]) outperform wrist sensors through sophisticated filtering algorithms. At the opposite end of the spectrum lie tools such as Welltory [8], for which independent studies report critically wide limits of agreement that preclude rigorous application. While the Garmin Elevate v4 demonstrated high test–retest reliability in the present study, its systematic bias and limits of agreement preclude its use as a direct substitute for ECG when precise HRV quantification is required. This finding aligns with recent evidence in trained athletes demonstrating that, whilst PPG-based devices yield good-to-excellent reliability, they incur higher measurement error and wider limits of agreement than ECG or chest-strap telemetry [63]. Consequently, replacing ECG with wrist-worn PPG devices for high-precision HRV assessment should be approached with caution. Under the conditions analysed, their use is constrained by a bias that limits agreement with ECG-derived HRV.

HRV monitoring is established as a practical strategy for evaluating daily autonomic balance [64,65]. Under this paradigm, implementing the Garmin Forerunner 955 for absolute HRV assessment via optical PRV introduces analytical challenges. Due to its proportional bias, the device tends to underestimate true ECG-derived rMSSD_HRV_ in individuals presenting higher vagal tone. Consequently, a healthy individual presenting elevated parasympathetic modulation could register a fictitious drop in PPG-derived PRV (rMSSD_PRV_) induced by measurement error rather than physiological variation. Following standard monitoring protocols, practitioners or active individuals might misinterpret this artifact as heightened fatigue or incomplete recovery. Therefore, while the device provides useful relative PRV trends, its measurement discrepancies compared to electrical HRV should be carefully considered when evaluating resting autonomic status in healthy adults.

This study presents certain limitations that must be acknowledged when interpreting the results. Firstly, the Health Snapshot feature operates within a closed ecosystem, acting as a ‘black box’ that prevents the export of raw waveform data to apply standardised interpolation filters on precise beat-to-beat intervals for PRV analysis. Secondly, ecological validity was compromised, given that strict control of respiratory rate [27] was indispensable to isolate instrumental error, thereby limiting the generalisability of the findings to spontaneous breathing conditions [66]. Finally, the homogeneity of the study sample constitutes another important limitation, as world-class elite athletes (rMSSD_HRV_ > 150 ms), a population in which the proportional bias between PRV and HRV detected in this study may be more pronounced, were not included. Furthermore, because all measurements were obtained within a single laboratory session, this study characterises intrasession test–retest repeatability rather than true longitudinal reliability. A key methodological consideration of the present study is its reliance on a highly controlled laboratory environment, including paced breathing and fixed postures. While this strict design was intentionally selected to eliminate motion artifacts and establish the true baseline measurement accuracy of the sensor, it does not reflect unconstrained, dynamic day-to-day conditions. Consequently, our findings are specific to standardized resting assessments, and future research is needed to explore the device’s accuracy across naturalistic free-living activities.

Future research should include larger and more diverse cohorts to enhance the external validity of these findings. Incorporating repeated measurements over consecutive days is essential to establish the true longitudinal tracking capability and generalisability of optical PRV estimates derived from the Health Snapshot feature. Furthermore, there is a clear imperative for metric transparency: assessing the direct impact of filtering algorithms [41] will be crucial once manufacturers permit the extraction of raw beat-to-beat intervals. It is also of great interest to develop multicentre comparisons that simultaneously evaluate ECG-derived HRV against various wearables, accounting for hardware and software disparities under identical postural protocols. Lastly, deeper investigation into tissue-related factors such as skin tone, capillary density, and arterial stiffness is vital to better understand their modulating effect on the bias amplification inherent to optical PRV technology [67,68].

## 5. Conclusions

The Garmin Forerunner 955 (Elevate v4) smartwatch demonstrates excellent concurrent validity and robust temporal reliability for measuring resting HR, establishing itself as a viable alternative to electrocardiography for basic haemodynamic variables.

Regarding PPG-derived PRV (rMSSD_PRV_), the device exhibits outstanding test–retest reliability (CV_SEM_ < 3.5%), allowing for the consistent short-term intrasession tracking of within-participant PRV trends. However, it lacks adequate concurrent validity against the gold standard. The marked proportional bias becomes increasingly more pronounced at higher levels of parasympathetic modulation, generating unacceptable individual discrepancies (ranging from −43% to +118% across positions). Consequently, although the device is useful for the recreational monitoring of relative trends, it is not bio statistically interchangeable with ECG for absolute resting HRV assessment and should be used with caution when evaluating healthy adults with high parasympathetic tone.

## Figures and Tables

**Figure 1:**
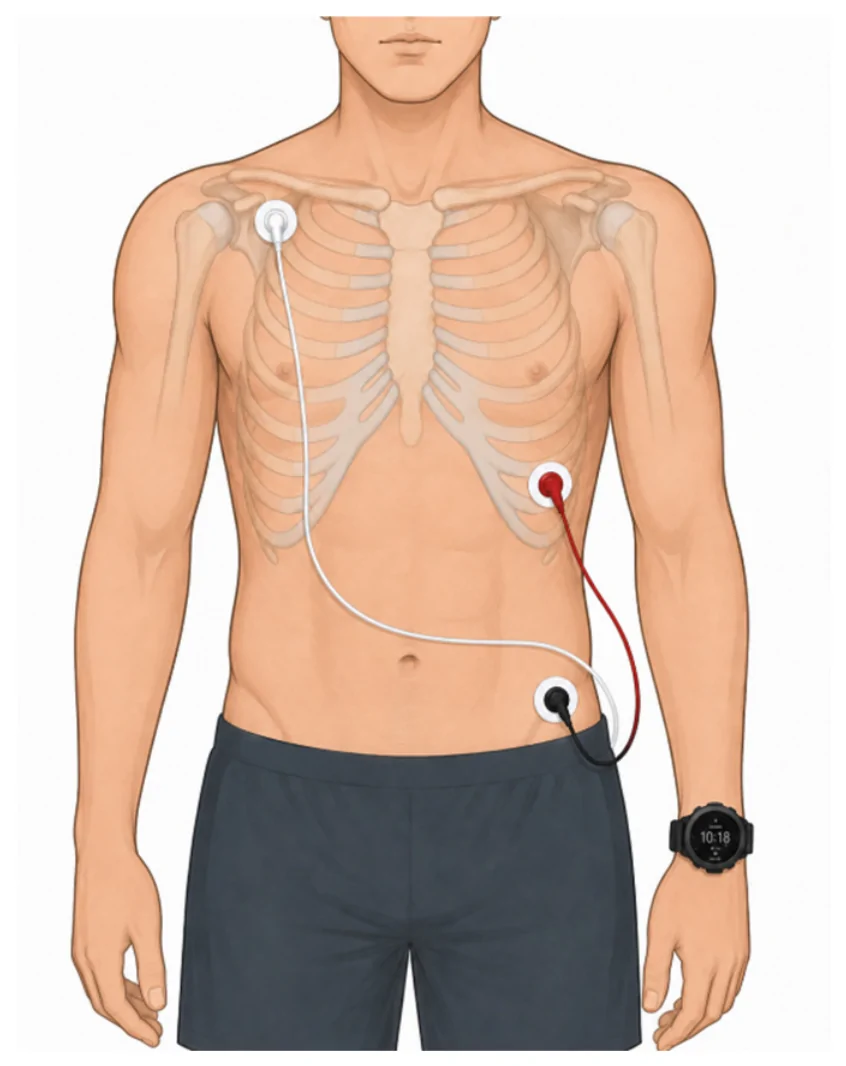
Anatomical placement of electrodes for electrocardiogram recording.

**Figure 2:**
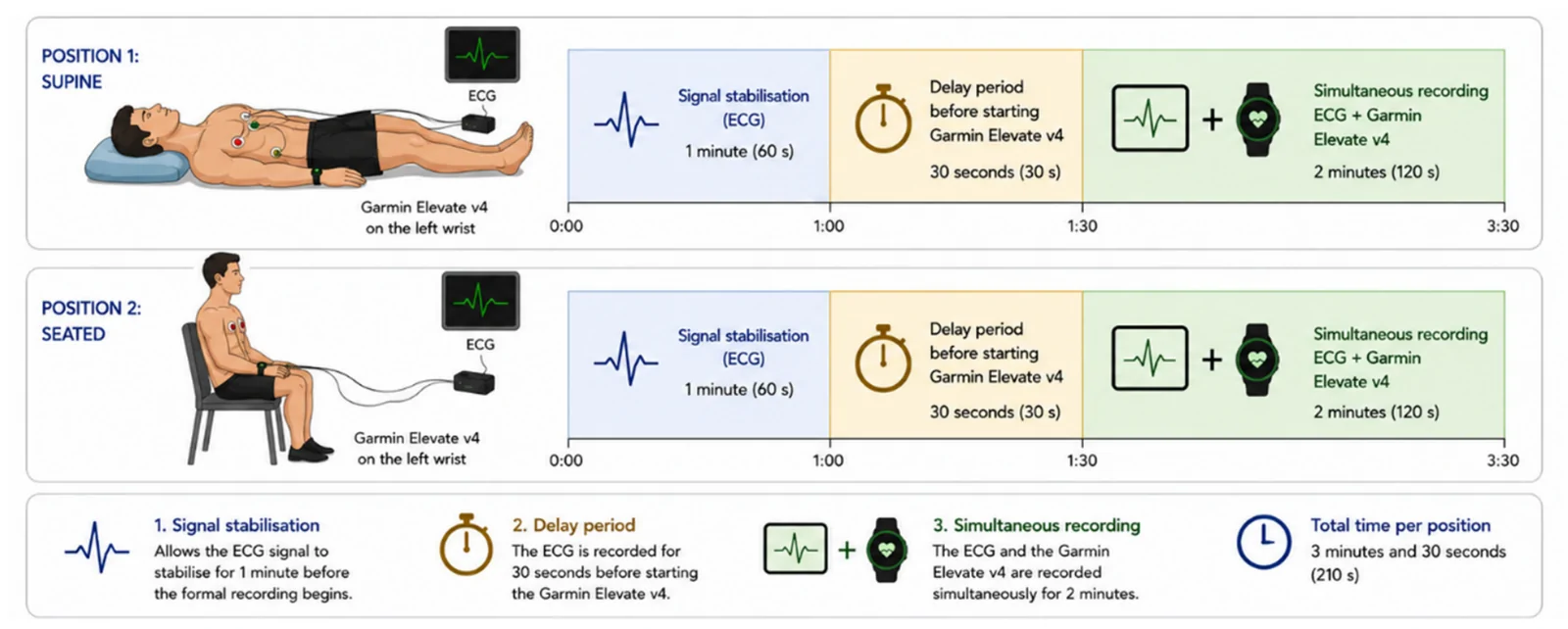
Experimental protocol design.

**Figure 3:**
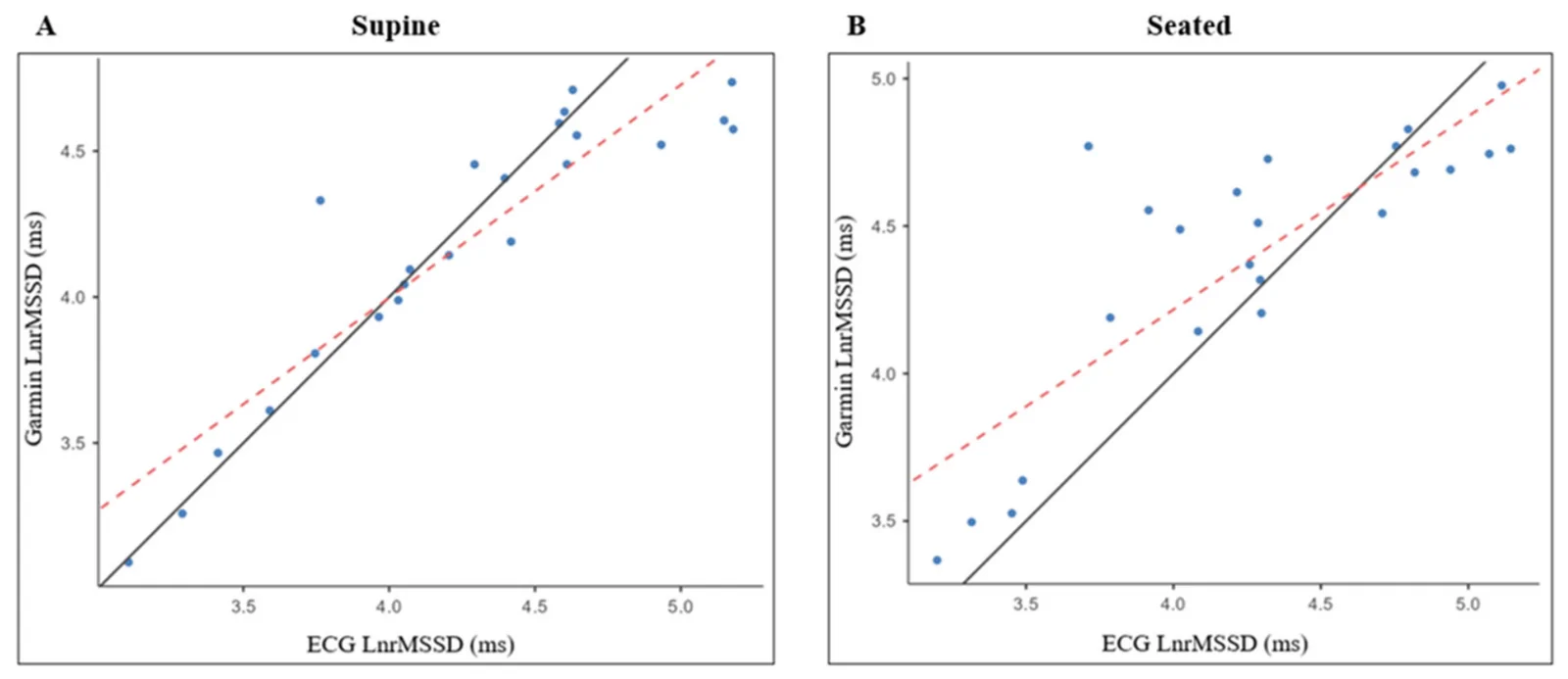
Correlation and concordance plots comparing Garmin PPG-derived PRV (LnrMSSD_PRV_) versus ECG-derived HRV (LnrMSSD_HRV_) in the supine (**A**) and seated (**B**) positions. Blue dots represent individual data points. The solid black line indicates the line of identity (y = x), and the dashed red line represents the linear association.

**Figure 4:**
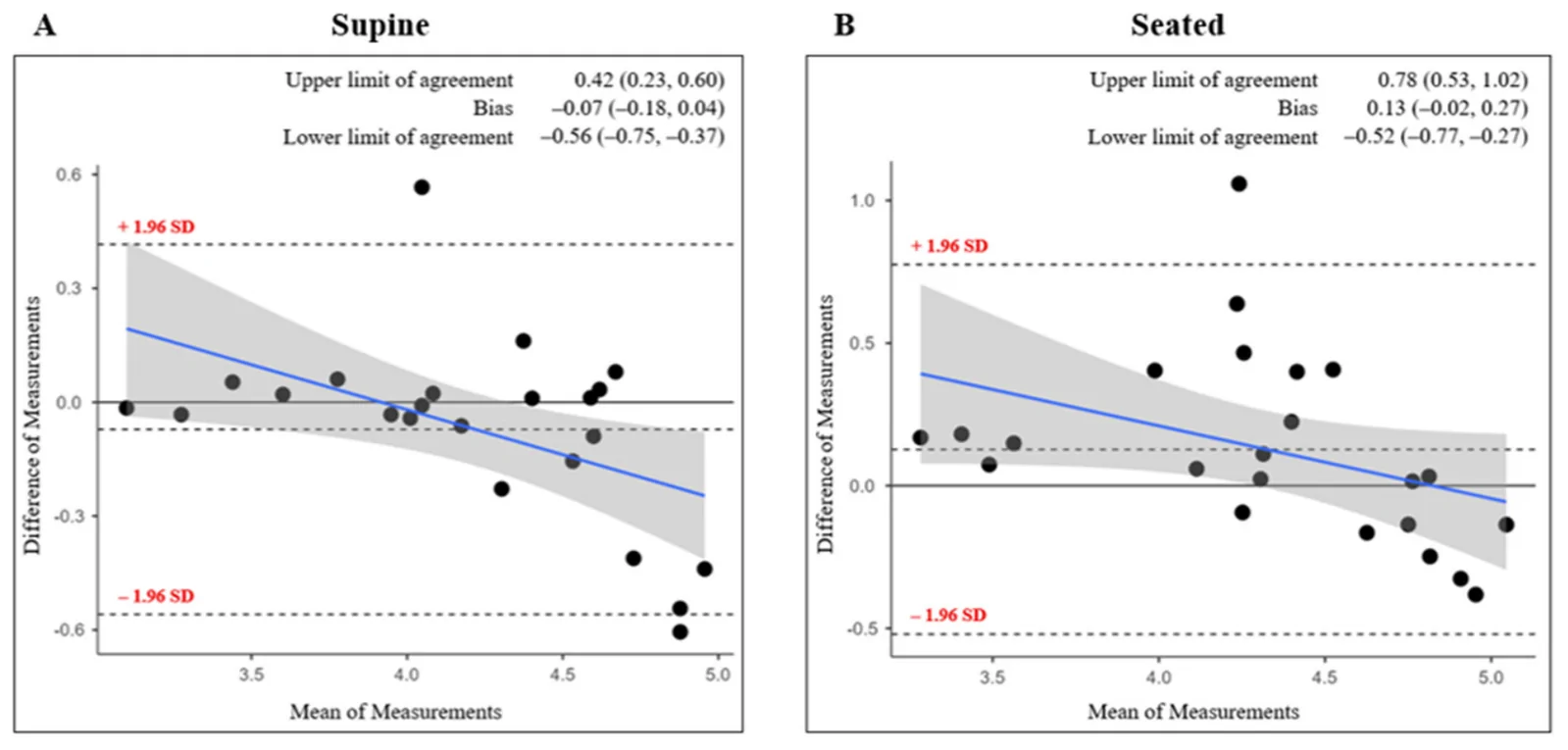
Bland–Altman plots assessing agreement in pulse rate variability (LnrMSSD). The graphs compare Garmin PPG-derived PRV (LnrMSSD_PRV_) against the reference ECG-derived HRV (LnrMSSD_HRV_) in the supine (**A**) and seated (**B**) positions. Black dots represent individual observations. The solid blue line indicates the fitted regression line, surrounded by the 95% confidence interval (shaded grey area). The horizontal lines represent the zero-bias line (solid line) and the mean difference (dashed line).

**Figure 5:**
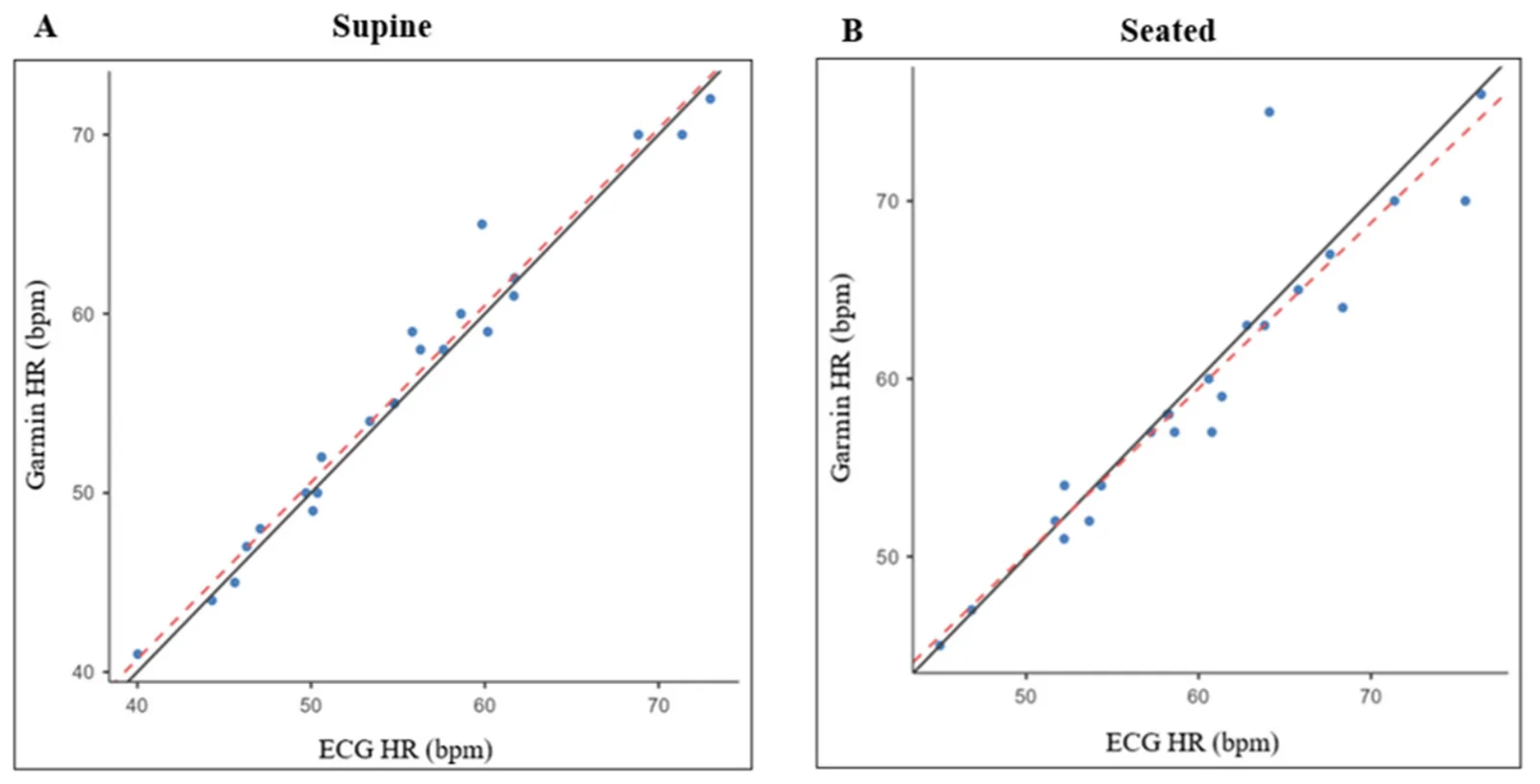
Correlation and concordance plots for HR. Comparison of values obtained via Garmin PPG versus ECG in the supine (**A**) and seated (**B**) positions. Blue dots represent individual data points. The solid black line indicates the line of identity (y = x), and the dashed red line represents the linear association.

**Figure 6:**
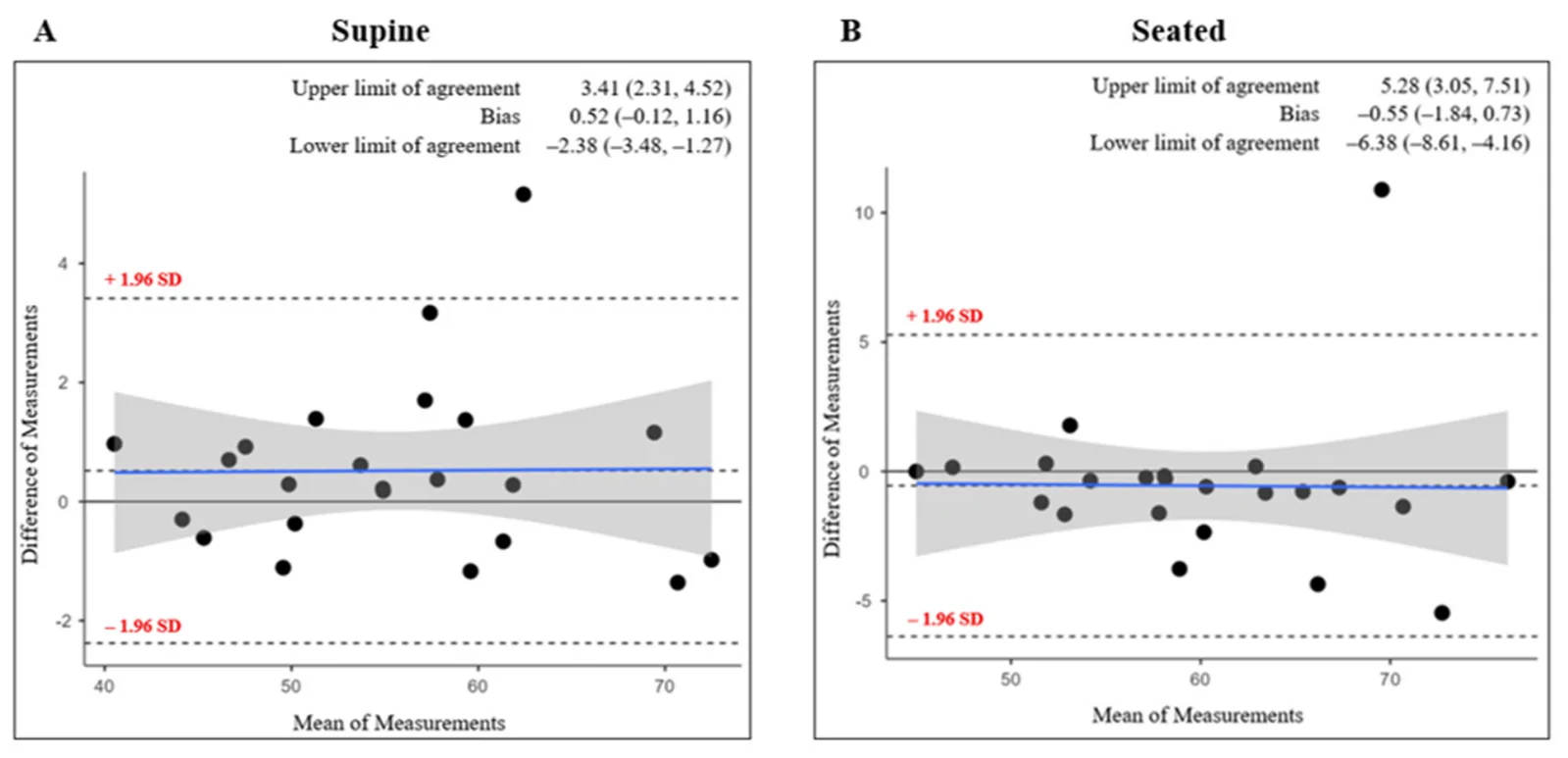
Bland–Altman plots assessing agreement in HR. The graphs compare Garmin PPG measurements against the reference ECG in the supine (**A**) and seated (**B**) positions. Black dots represent individual observations. The solid blue line indicates the fitted regression line, surrounded by the 95% confidence interval (shaded grey area). The horizontal lines represent the zero-bias line (solid line) and the mean difference (dashed line).

**Table 1:** Descriptive statistics of participants (mean ± SD).

Characteristics	Males (*n* = 12)	Females (*n* = 11)	All (*n* = 23)
Age (y)	30.42 ± 8.23	28.09 ± 5.28	29.30 ± 6.92
Weight (kg)	74.73 ± 9.70	62.77 ± 7.17	69.01 ± 10.38
Height (m)	1.75 ± 0.07	1.62 ± 0.07	1.69 ± 0.10
BMI (kg/m^−2^)	24.23 ± 1.95	23.83 ± 2.05	24.04 ± 1.96
Breathing frequency (bpm)	14.08 ± 1.56	14.09 ± 1.45	14.09 ± 1.47

BMI, body mass index; bpm, breaths per minute; kg, kilograms; m, meters; *n*, sample number; SD, standard deviation; y, years.

**Table 2:** Statistical concordance and correlation analysis comparing Garmin PPG-derived PRV (LnrMSSD_PRV_) with reference ECG-derived HRV (LnrMSSD_HRV_) in varying positions.

Position	Device	Descriptive	*r* (95% CI)	*p^A^*	CCC (95% CI)	*p^B^*	*C_b_*
Supine	Garmin PPG	4.18 ± 0.47	0.92 (0.81, 0.96)	<0.01	0.89 (0.80, 0.97)	<0.01	0.97
	ECG	4.25 ± 0.59					
Seated	Garmin PPG	4.39 ± 0.47	0.83 (0.63, 0.93)	<0.01	0.78 (0.64, 0.93)	<0.01	0.95
	ECG	4.26 ± 0.59					

*C_b_*, bias correction factor; CCC, concordance correlation coefficient; CI, confidence interval; ECG, electrocardiogram; LnrMSSD, natural logarithm of the root mean square of successive differences; *p^A^*, significance Pearson correlation coefficient; *p^B^*, significance concordance correlation coefficient; PPG, photoplethysmography.

**Table 3:** Relative and absolute test–retest reliability indices, measurement error metrics, and minimal detectable change for PPG-derived PRV (LnrMSSD_PRV_) and ECG-derived HRV (LnrMSSD_HRV_) across postural conditions.

Position	Device	ICC (95% CI)	MAE	MAPE	SEM	CV_SEM_ (%)	MDC
Supine	Garmin PPG	0.84 (0.67, 0.93)	0.19	4.72	0.13	3.07	0.36
	ECG	0.84 (0.66, 0.93)	0.23	5.55	0.18	4.28	0.50
Seated	Garmin PPG	0.79 (0.57, 0.91)	0.21	4.73	0.15	3.43	0.42
	ECG	0.96 (0.90, 0.98)	0.14	3.51	0.09	2.09	0.25

CI, confidence interval; CV, coefficient of variation; ECG, electrocardiogram; ICC, intraclass correlation coefficient; LnrMSSD, natural logarithm of the root mean square of successive differences; MAE, mean absolute error; MAPE, mean absolute percentage error; MDC, minimal detectable change; PPG, photoplethysmography; SEM, standard error of measurement.

**Table 4:** Statistical concordance and correlation analysis of HR comparing Garmin PPG with reference ECG in varying positions.

Position	Device	Descriptive	*r* (95% CI)	*p^A^*	CCC (95% CI)	*p^B^*	*C_b_*
Supine	Garmin PPG	55.83 ± 8.55	0.99 (0.96, 0.99)	<0.01	0.98 (0.97, 0.99)	<0.01	0.99
	ECG	55.31 ± 8.54					
Seated	Garmin PPG	59.74 ± 8.25	0.94 (0.85, 0.97)	<0.01	0.93 (0.88, 0.99)	<0.01	0.99
	ECG	60.29 ± 8.30					

*C_b_*, bias correction factor; CCC, concordance correlation coefficient; CI, confidence interval; ECG, electrocardiogram; HR, heart rate; *p^A^*, significance Pearson correlation coefficient; *p^B^*, significance concordance correlation coefficient; PPG, photoplethysmography.

**Table 5:** Relative and absolute test–retest reliability indices, measurement error metrics, and minimal detectable change for HR across postural conditions.

Position	Device	ICC (95% CI)	MAE	MAPE	SEM	CV_SEM_ (%)	MDC
Supine	Garmin PPG	0.92 (0.83, 0.97)	3.78	6.33	2.41	4.19	6.68
	ECG	0.93 (0.83, 0.97)	4.31	7.37	2.37	4.12	6.57
Seated	Garmin PPG	0.92 (0.83, 0.97)	3.35	5.23	2.10	3.43	5.82
	ECG	0.93 (0.85, 0.97)	3.18	5.10	1.96	3.18	5.43

CI, confidence interval; CV, coefficient of variation; ECG, electrocardiogram; HR, heart rate; ICC, intraclass correlation coefficient; MAE, mean absolute error; MAPE, mean absolute percentage error; MDC, minimal detectable change; MD, mean difference; PPG, photoplethysmography; SEM, standard error of measurement.

## Data Availability

The datasets generated from the current study are available from the corresponding author on reasonable request.

## Supplementary Materials

The following supporting information can be downloaded at https://www.mdpi.com/article/10.3390/s26206549/s1, Table S1: Automatic artifact correction rates (Kubios) for seated condition (Trial 1); Table S2: Automatic artifact correction rates (Kubios) for seated condition (Trial 2); Table S3: Automatic artifact correction rates (Kubios) for supine condition (Trial 1); Table S4: Automatic artifact correction rates (Kubios) for supine condition (Trial 2).

## Abbreviations

The following abbreviations are used in this manuscript:

HR	Heart rate
HRV	Heart rate variability
ECG	Electrocardiogram
rMSSD	Root mean square of successive differences
R-R intervals	Temporal fluctuation between consecutive heartbeats
ANS	Autonomic nervous system
PPG	Photoplethysmography
PRV	Pulse rate variability
GRRAS	Guidelines for Reporting Reliability and Agreement Studies
RSA	Respiratory sinus arrhythmia
CS5	Central Subclavicular 5
LnrMSSD	Natural logarithmic transformation root mean square of successive differences
SD	Standard deviation
*r*	Pearson correlation coefficient
*C_b_*	Bias correction factor
CCC, *p_c_*	Lin’s Concordance Correlation Coefficient
LoA	Limits of agreement
ICC	Intraclass correlation coefficient
SEM	Standard error of measurement
CV_SEM_	Coefficient of variation of standard error of measurement
MDC	Minimal detectable change
MAE	Mean absolute error
MAPE	Mean absolute percentage error
CI	Confidence intervals
