# Supplementary material for: Pulse vs. R-R Interval: Validity and Reliability of the Garmin Elevate v4 Sensor for Ultra-Short HR and HRV Monitoring in Healthy Adults
Source: Sensors (Basel). 2026 Oct 15;26(20):6549. doi: 10.3390/s26206549 (PMC13709044; doi:10.3390/s26206549)
Supplement: Supplementary file 1 [file sensors-26-06549-s001.zip › sensors-4571221-supplementary.pdf]

Supplementary Material

Table S1. Automatic artifact correction rates (Kubios) for seated condition (Trial 1).

Code	Beats total	Beats corrected	Beats corrected (%)
Participant 01	122	0	0
Participant 02	125	0	0
Participant 03	86	0	0
Participant 04	130	0	0
Participant 05	139	0	0
Participant 06	95	0	0
Participant 07	105	0	0
Participant 08	115	0	0
Participant 09	113	0	0
Participant 10	103	0	0
Participant 11	155	2	1.29
Participant 12	115	0	0
Participant 13	131	0	0
Participant 14	154	0	0
Participant 15	152	0	0
Participant 16	138	0	0
Participant 17	134	0	0
Participant 18	114	0	0
Participant 19	125	0	0
Participant 20	140	0	0
Participant 21	136	0	0
Participant 22	128	0	0
Participant 23	135	0	0
**Mean**	125.65	0.09	0.00
**SD**	18.09	0.42	0.00

SD, standard deviation.

Table S2. Automatic artifact correction rates (Kubios) for seated condition (Trial 2).

Code	Beats total	Beats corrected	Beats corrected (%)
Participant 01	115	0	0
Participant 02	122	0	0
Participant 03	90	0	0
Participant 04	117	0	0
Participant 05	116	0	0
Participant 06	94	0	0
Participant 07	104	0	0
Participant 08	116	0	0
Participant 09	109	0	0
Participant 10	104	0	0
Participant 11	155	0	0
Participant 12	109	0	0
Participant 13	123	0	0
Participant 14	143	0	0
Participant 15	151	0	0
Participant 16	128	0	0
Participant 17	131	0	0
Participant 18	108	0	0

Participant 19	122	0	0
Participant 20	137	0	0
Participant 21	135	0	0
Participant 22	121	0	0
Participant 23	125	0	0
**Mean**	120.65	0.00	0.00
**SD**	16.50	0.00	0.00

SD, standard deviation.

Table S3. Automatic artifact correction rates (Kubios) for supine condition (Trial 1).

Code	Beats total	Beats corrected	Beats corrected (%)
Participant 01	107	0	0
Participant 02	120	0	0
Participant 03	82	0	0
Participant 04	139	0	0
Participant 05	122	0	0
Participant 06	88	0	0
Participant 07	103	0	0
Participant 08	96	0	0
Participant 09	123	0	0
Participant 10	106	0	0
Participant 11	151	0	0
Participant 12	106	0	0
Participant 13	110	0	0
Participant 14	149	0	0
Participant 15	150	0	0
Participant 16	111	0	0
Participant 17	145	0	0
Participant 18	104	0	0
Participant 19	94	0	0
Participant 20	129	0	0
Participant 21	127	0	0
Participant 22	109	2	1.83
Participant 23	127	0	0
**Mean**	117.30	0.09	0.00
**SD**	20.05	0.42	0.00

SD, standard deviation.

Table S4. Automatic artifact correction rates (Kubios) for supine condition (Trial 2).

Code	Beats total	Beats corrected	Beats corrected (%)
Participant 01	101	0	0
Participant 02	113	0	0
Participant 03	80	0	0
Participant 04	120	0	0
Participant 05	111	0	0
Participant 06	88	0	0
Participant 07	94	0	0
Participant 08	100	0	0
Participant 09	107	0	0
Participant 10	100	0	0
Participant 11	136	0	0
Participant 12	79	1	1.27

Participant 13	110	0	0
Participant 14	143	0	0
Participant 15	146	0	0
Participant 16	109	0	0
Participant 17	117	2	1.71
Participant 18	94	4	4.26
Participant 19	120	0	0
Participant 20	115	0	0
Participant 21	123	0	0
Participant 22	101	0	0
Participant 23	123	0	0
**Mean**	110.00	0.30	0.00
**SD**	17.73	0.93	0.00

SD, standard deviation.
